# Supplementary material for: Discovery of human ACE2 variants with altered recognition by the SARS-CoV-2 spike protein
Source: PLoS One. 2021 May 12;16(5):e0251585. doi: 10.1371/journal.pone.0251585 (PMC8115845; doi:10.1371/journal.pone.0251585)
Supplement: S1 Fig — ACE2’s C-terminus was fused to a Myc epitope tag and the Aga2 native yeast surface protein on yeast cell wall. ACE2 display was quantified using an anti-myc chicken IgY that is detected using an Alexa488-conjugated anti-chicken goat IgG as secondary label (not depicted). Binding of yeast-displayed ACE2 to His6-tagged SARS-CoV-2 spike RBD was detected via incubation with Alexa647-conjugated anti-His6 mouse IgG. Both ACE2 display and ACE2 binding to spike RBD were measured by flow cytometry. Each yeast cell displays up to 104 copies of a single ACE2 variant on its surface. (PDF) [file pone.0251585.s001.pdf]

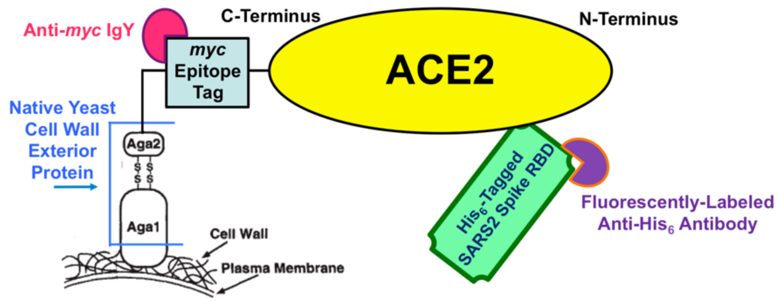

Supporting Figure 1. Yeast display schematic. ACE2's C-terminus was fused to a Myc epitope tag and the Aga2 native yeast surface protein on yeast cell wall. ACE2 display was quantified using an anti-*myc* chicken IgY that is detected using an Alexa488-conjugated anti-chicken goat IgG as secondary label (not depicted). Binding of yeast-displayed ACE2 to His_6-tagged SARS-CoV-2 spike RBD was detected via incubation with Alexa647-conjugated anti-His_6 mouse IgG. Both ACE2 display and ACE2 binding to spike RBD were measured by flow cytometry. Each yeast cell displays up to 10^4 copies of a single ACE2 variant on its surface.
