# Supplementary material for: Discovery of human ACE2 variants with altered recognition by the SARS-CoV-2 spike protein
Source: PLoS One. 2021 May 12;16(5):e0251585. doi: 10.1371/journal.pone.0251585 (PMC8115845; doi:10.1371/journal.pone.0251585)
Supplement: S2 Fig — Sort gate (black polygon) was defined to enrich the top twenty percent of myc-positive (ACE2 displaying) yeast. The X-axis denotes Alexa488 fluorescence (ACE2 display) and the Y-axis denotes Alexa647 fluorescence (ACE2 binding to spike protein). The plot depicts dots for approximately 1.5*106 yeast cells. The library was incubated with 150 nM spike RBD prior to sorting. The high density (red) oval at the lower left primarily contains yeast that have not been induced. For biological reasons that are poorly understood, even homogeneous populations of yeast carrying identical display plasmids, i.e., wild type ACE2, feature 25% or greater cells that do not display any protein. (PDF) [file pone.0251585.s002.pdf]

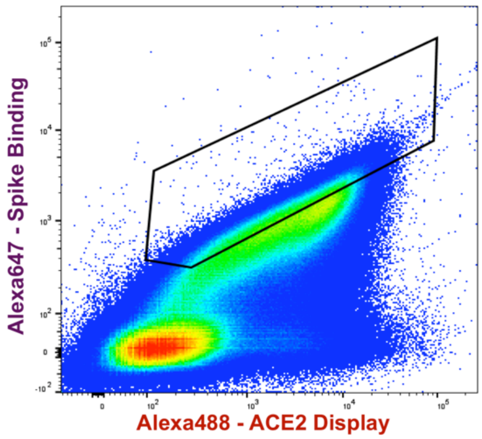

Supporting Figure 2. Dot plot for FACS of random mutant ACE2 library. Sort gate (black polygon) was defined to enrich the top twenty percent of *myc*-positive (ACE2 displaying) yeast. The X-axis denotes Alexa488 fluorescence (ACE2 display) and the Y-axis denotes Alexa647 fluorescence (ACE2 binding to spike protein). The plot depicts dots for approximately $1.5*10^6$ yeast cells. The library was incubated with 150 nM spike RBD prior to sorting. The high density (red) oval at the lower left primarily contains yeast that have not been induced. For biological reasons that are poorly understood, even homogeneous populations of yeast carrying identical display plasmids, i.e., wild type ACE2, feature 25% or greater cells that do not display any protein.
