# Supplementary material for: Discovery of human ACE2 variants with altered recognition by the SARS-CoV-2 spike protein
Source: PLoS One. 2021 May 12;16(5):e0251585. doi: 10.1371/journal.pone.0251585 (PMC8115845; doi:10.1371/journal.pone.0251585)
Supplement: S4 Fig — The X-axes denote Alexa488 fluorescence (ACE2 display) and the Y-axes denote Alexa647 fluorescence (ACE2 binding to spike protein). The plots depict dots for approximately 3*104 yeast cells. The yeast were incubated with spike RBD at concentrations noted in figure prior to analysis. For biological reasons that are poorly understood, even homogeneous populations of yeast carrying identical display plasmids, i.e., wild-type ACE2, feature 25% or greater cells (lower left region of plots) that do not display any protein. (PDF) [file pone.0251585.s004.pdf]

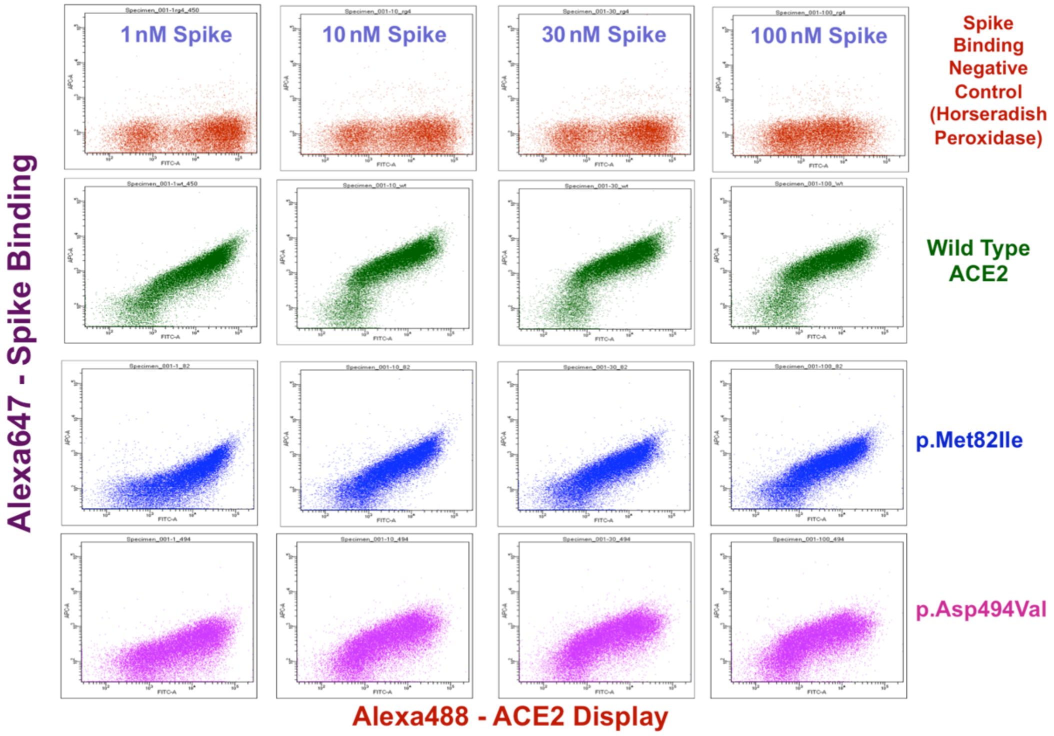

Supporting Figure 4. Representative flow cytometry dot plots corresponding to binding signal results presented in Figure 2. The X-axes denote Alexa488 fluorescence (ACE2 display) and the Y-axes denote Alexa647 fluorescence (ACE2 binding to spike protein). The plots depict dots for approximately $3*10^4$ yeast cells. The yeast were incubated with spike RBD at concentrations noted in figure prior to analysis. For biological reasons that are poorly understood, even homogeneous populations of yeast carrying identical display plasmids, i.e., wild-type ACE2, feature 25% or greater cells (lower left region of plots) that do not display any protein.
