# Supplementary material for: Discovery of human ACE2 variants with altered recognition by the SARS-CoV-2 spike protein
Source: PLoS One. 2021 May 12;16(5):e0251585. doi: 10.1371/journal.pone.0251585 (PMC8115845; doi:10.1371/journal.pone.0251585)
Supplement: S5 Fig — The X-axes denote Alexa647 fluorescence (ACE2 binding to spike protein), and the Y-axes denote the number of yeast cells. Each histogram represents approximately 3*104 yeast cells. The yeast were incubated with spike RBD at concentrations noted in the figure prior to analysis. For biological reasons that are poorly understood, even homogeneous populations of yeast carrying identical display plasmids, i.e., wild-type ACE2, feature 25% or greater cells (leftmost yeast in ACE2 histograms) that do not display any protein. (PDF) [file pone.0251585.s005.pdf]

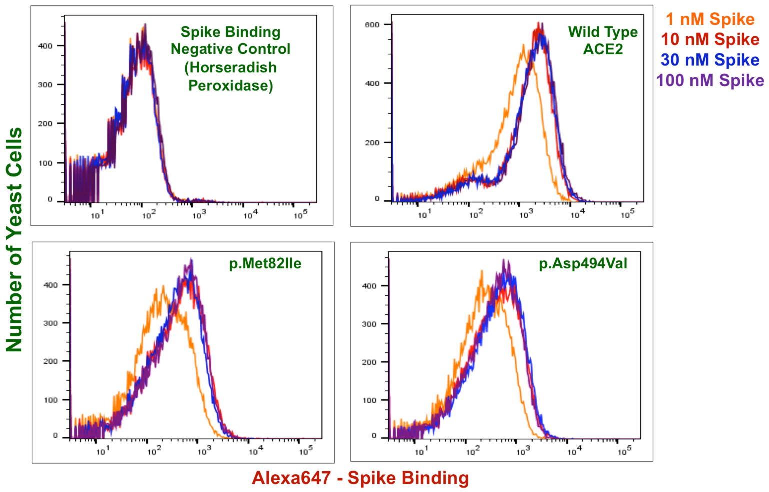

Supporting Figure 5. Representative flow cytometry histogram overlays corresponding to results presented in Figure 2. The X-axes denote Alexa647 fluorescence (ACE2 binding to spike protein), and the Y-axes denote the number of yeast cells. Each histogram represents approximately $3*10^4$ yeast cells. The yeast were incubated with spike RBD at concentrations noted in the figure prior to analysis. For biological reasons that are poorly understood, even homogeneous populations of yeast carrying identical display plasmids, i.e., wild-type ACE2, feature 25% or greater cells (leftmost yeast in ACE2 histograms) that do not display any protein.
