# Supplementary material for: Discovery of human ACE2 variants with altered recognition by the SARS-CoV-2 spike protein
Source: PLoS One. 2021 May 12;16(5):e0251585. doi: 10.1371/journal.pone.0251585 (PMC8115845; doi:10.1371/journal.pone.0251585)
Supplement: S6 Fig — The X-axes denote Alexa488 fluorescence (ACE2 display) and Y-axes denote Alexa647 fluorescence (ACE2 binding to spike protein). The plots depict dots for approximately 3*104 yeast cells. The dark blue line denotes upper limit of Alexa488 signal (ACE2 display) for wild-type ACE2. The yeast were incubated with 5 nM spike RBD prior to flow cytometric analysis. For biological reasons that are poorly understood, even homogeneous populations of yeast carrying identical display plasmids, i.e., wild-type ACE2, feature 25% or greater cells (lower left region of plots) that do not display any protein. (PDF) [file pone.0251585.s006.pdf]

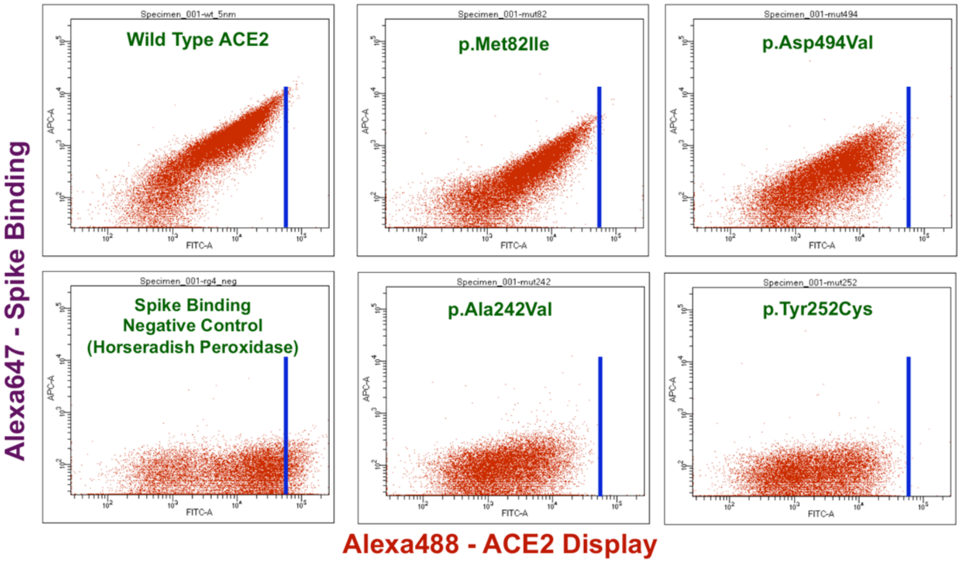

Supporting Figure 6. Flow cytometry dot plots for wild type and selected SNV ACE2s. The X-axes denote Alexa488 fluorescence (ACE2 display) and Y-axes denote Alexa647 fluorescence (ACE2 binding to spike protein). The plots depict dots for approximately $3*10^4$ yeast cells. The dark blue line denotes upper limit of Alexa488 signal (ACE2 display) for wild-type ACE2. The yeast were incubated with 5 nM spike RBD prior to flow cytometric analysis. For biological reasons that are poorly understood, even homogeneous populations of yeast carrying identical display plasmids, i.e., wild-type ACE2, feature 25% or greater cells (lower left region of plots) that do not display any protein.
